# Sugar infusion into trees: A novel method to study tree carbon relations and its regulations

**DOI:** 10.3389/fpls.2023.1142595

**Published:** 2023-02-23

**Authors:** Yan-Li Zhang, Yue Yang, Matthias Saurer, Marcus Schaub, Arthur Gessler, Marco M. Lehmann, Andreas Rigling, Marco Walser, Beat Stierli, Noureddine Hajjar, Daniel Christen, Mai-He Li

**Affiliations:** ^1^ College of Ecology and Environment, Hainan University, Haikou, China; ^2^ Forest Dynamics, Swiss Federal Institute for Forest, Snow and Landscape Research WSL, Birmensdorf, Switzerland; ^3^ Department of Environmental Systems Science, Institute of Terrestrial Ecosystems, ETH Zürich, Zürich, Switzerland; ^4^ Forest Soils and Biogeochemistry, Swiss Federal Institute for Forest, Snow and Landscape Research WSL, Birmensdorf, Switzerland; ^5^ Key Laboratory of Geographical Processes and Ecological Security in Changbai Mountains, Ministry of Education, School of Geographical Sciences, Northeast Normal University, Changchun, Jilin, China; ^6^ College of Life Science, Hebei University, Baoding, Hebei, China

**Keywords:** ^13^C-labelled glucose, exogenous sugars, carbon limitation, carbon physiology, methodology, tree infusion

## Abstract

Many carbon-related physiological questions in plants such as carbon (C) limitation or starvation have not yet been resolved thoroughly due to the lack of suitable experimental methodology. As a first step towards resolving these problems, we conducted infusion experiments with bonsai trees (*Ficus microcarpa*) and young maple trees (*Acer pseudoplatanus*) in greenhouse, and with adult Scots pine trees (*Pinus sylvestris*) in the field, that were “fed” with ^13^C-labelled glucose either through the phloem or the xylem. We then traced the ^13^C-signal in plant organic matter and respiration to test whether trees can take up and metabolize exogenous sugars infused. Ten weeks after infusion started, xylem but not phloem infusion significantly increased the δ^13^C values in both aboveground and belowground tissues of the bonsai trees in the greenhouse, whereas xylem infusion significantly increased xylem δ^13^C values and phloem infusion significantly increased phloem δ^13^C values of the adult pines in the field experiment, compared to the corresponding controls. The respiration measurement experiment with young maple trees showed significantly increased δ^13^C-values in shoot respired CO_2_ at the time of four weeks after xylem infusion started. Our results clearly indicate that trees do translocate and metabolize exogenous sugars infused, and because the phloem layer is too thin, and thus xylem infusion can be better operated than phloem infusion. This tree infusion method developed here opens up new avenues and has great potential to be used for research on the whole plant C balance and its regulation in response to environmental factors and extreme stress conditions.

## 1 Introduction

Trees rely on photosynthesis to gain carbon, which is a core resource allocated among different tissues to grow, reproduce, defend, and maintain metabolic functions (Körner, 2006; Maguire and Kobe, 2015; Hartmann and Trumbore, 2016). Photosynthetic carbon may be passively (Sala et al., 2010) or actively (Li et al., 2018) stored in the form of sugars and starch as nonstructural carbohydrates (NSC) in response to environmental stress, thus the plant tissue NSC levels reflect the balance between C-gain (or supply) and C-utilization (or demand) (Chapin et al., 1990; Kozlowski, 1992; Li et al., 2002; Hoch et al., 2003; Körner, 2003; Dietze et al., 2014; O’Brien et al., 2014; Hartmann and Trumbore, 2016; Martínez-Vilalta et al., 2016; Sapes et al., 2021). If plant’s C demand exceeds the C supply, the plant’s C storage will be depleted with time and the plant may suffer from a carbon deficit, which may result in decreasing growth rate and increasing mortality of plants.

In the debate about either the alpine treeline formation or the drought-induced forest mortality, carbon shortage or carbon starvation has been proposed and are still being debated as the underlying physiological mechanisms. Studies based on natural experiments suggested carbon source limitation (Wardle, 1993; Li et al., 2002; Li et al., 2008a; Li et al., 2008b; Li et al., 2018; Wang et al., 2021) or growth sink limitation (Körner, 1998; Hoch and Körner, 2009; Harsch and Bader, 2011) as the possible physiological mechanisms for tree growth under low temperature at the alpine treelines. Similarly, hydraulic failure and carbon starvation have been proposed as possible physiological mechanisms to explain the water deficit-caused forest dieback (McDowell et al., 2008; McDowell et al., 2011; Mcdowell et al., 2016; Choat et al., 2018). The carbon starvation or limitation hypothesis assumes that mortality of drought- or low temperature-stressed trees occurs because trees’ carbon production of photosynthesis cannot meet its minimum demand for survival (Li et al., 2008b; McDowell and Sevanto, 2010). Measurements of tissue NSC levels reveal, however, inconsistent results. For example, as Hoch and Körner (2003) did not find any carbon shortage in treeline trees, other studies clearly indicated a carbon limitation in roots of plants at their upper limits (Li et al., 2008a; Li et al., 2008b; Genet et al., 2011; Li et al., 2018; Wang et al., 2021) and under water deficit (Galvez et al., 2013; Sevanto and Dickman, 2015; Hagedorn et al., 2016; Joseph et al., 2020; Schönbeck et al., 2020). Moreover, in both the treeline and drought cases, carbon limitation may occur with growth limitation (treeline) or hydraulic failure (drought) together (Adams et al., 2017), and thus, we actually don’t know which one is the primary and which is the following secondary mechanisms leading to mortality. If non-structural carbohydrates could be introduced into stressed trees, and if trees can accept, distribute and metabolize such exogenous sugars added, it is then possible to distinguish carbon limitation from growth limitation or hydraulic failure.

We therefore ask whether plants, analogously to nutritionally deficit sick humans and animals, can accept and use exogenous sugars infused, because sugars in the nature are merely produced by plant photosynthesis (endogenous sugars)? If yes, we, then, can develop a new methodology (i.e. exogenous sugar addition) to open up new avenues for research on the whole plant carbon balance and its regulations, and potentially improve vitality of historic and landmark trees suffering from severe damage in the practice.

Since more than 50 years, some previous activities have tried to develop plant injection methods and techniques to inject chemical substances into tree’s stem for pests control (Feng, 1972; Feng, 1979; Eggers et al., 2005; Feng and Ji, 2009; Zeng et al., 2010; Hijaz et al., 2020), which has been recently reviewed by Li and Nangong (2022). Injection of plant nutrients (Grabau et al., 1986; Schon and Blevins, 1987) and hormones (Philipson, 1985; Ross and Bower, 1991; Eriksson et al., 1998; Smith, 1998; Kong et al., 2008) for growth regulations has also been applied. Other authors demonstrated the possibility of supplying albino corn plants (Spoehr, 1942) and tomato plants (Went and Carter, 1948) with sucrose, or feeding trees (*Picea abies* [L.] Karst) with amino acids (Gessler et al., 2003) through the cut ends of the leaves.

Overall, there are only a few studies worldwide that injected (fast, short time treatment) or infused (slow, longer time treatment) sugars into plant stems, and these very limited experiments concentrated only on a few agricultural species, i.e. soybean (Hayati et al., 1995; Abdin et al., 1998; Zhou et al., 2000), corn plants (*Zea mays* L.) (Boyle et al., 1991a; Boyle et al., 1991b; Ma et al., 1994; Zhou and Smith, 1996; Zhou et al., 1997), sweet and grain sorghum (Tarpley et al., 1994), and chickpea (*Cicer arietinum* L.) (Khan et al., 2017). They generally found that sugar injection or infusion increased yield but decreased plant photosynthesis. On the other hand, probably due to the hardness and rigidity of wood and bark, the possibility and methodology of sugar infusion or injection with woody plants have rarely been studied, tested only in *Quercus alba* (McLaughlin et al., 1980) using the circumferential trough technique (Auerbach et al., 1964), *Citrus unshiu* (Iglesias et al., 2003) with a low-pressure injection system (Iglesias et al., 2001), and in *Quercus virginiana* (Martinez-Trinidad et al., 2009) using the so-called macro-injection system (Eggers et al., 2005). They did not trace the transport and allocation of the injected/infused sugars within a tree, and thus, we actually do not know how the exogenous sugars added are transported and allocated in plants.

It is well known that water movement in xylem is unidirectional upwards from roots to aerial parts of trees, whereas the movement of photosynthetic carbon products in phloem is bidirectional both downwards and upwards from source (leaves or storage) to sink. As many studies indicated that carbon shortage occurs mainly in roots (Li et al., 2008a; Li et al., 2008b; Genet et al., 2011; Galvez et al., 2013; Sevanto and Dickman, 2015; Hagedorn et al., 2016; Li et al., 2018; Joseph et al., 2020; Schönbeck et al., 2020; Wang et al., 2021) mentioned above, it is thus reasonable that sugar addition into phloem may be more effective than into xylem of stressed trees, to improve the root carbon supply and balance.

We therefore, carried out the present pilot study to test whether trees take up, translocate and metabolize infused exogenous sugars. Using common infusion method and equipment for human and animals, we experimentally tested to infuse ^13^C-labelled glucose through either phloem or xylem into bonsai trees (*Ficus microcarpa*) in greenhouse, and also into adult *Pinus sylvestris* trees in the field in 2021. Three and ten weeks after infusion started, we took samples to analyze the δ^13^C values in organic matter. We hypothesize that xylem infusion increases the δ^13^C values in leaves and wood above the infusion point due to unidirectional upward transport in xylem, whereas phloem infusion increases the overall δ^13^C values of plants due to bidirectional movement in phloem (Hypothesis I). This, in other words, means that trees do take up infused exogenous sugars. Moreover, we expect that xylem infusion will works better than phloem infusion (Hypothesis II) because the latter may be very difficult to be operated due to the very thin phloem layer. Based on the results gained above in 2021, we conducted a third experiment with young *Acer pseudoplatanus* trees in 2022. We first infused the trees with ^13^C-labelled glucose, and then measured the shoot dark respiration four weeks after infusion started, to test our third hypothesis that plants use the infused exogenous sugars for respiration (Hypothesis III) to provide energy for physiological processes and growth of trees.

## 2 Methodology development: Materials, equipment, and methods

### 2.1 Greenhouse experiment with bonsai plants

**Figure 1:**
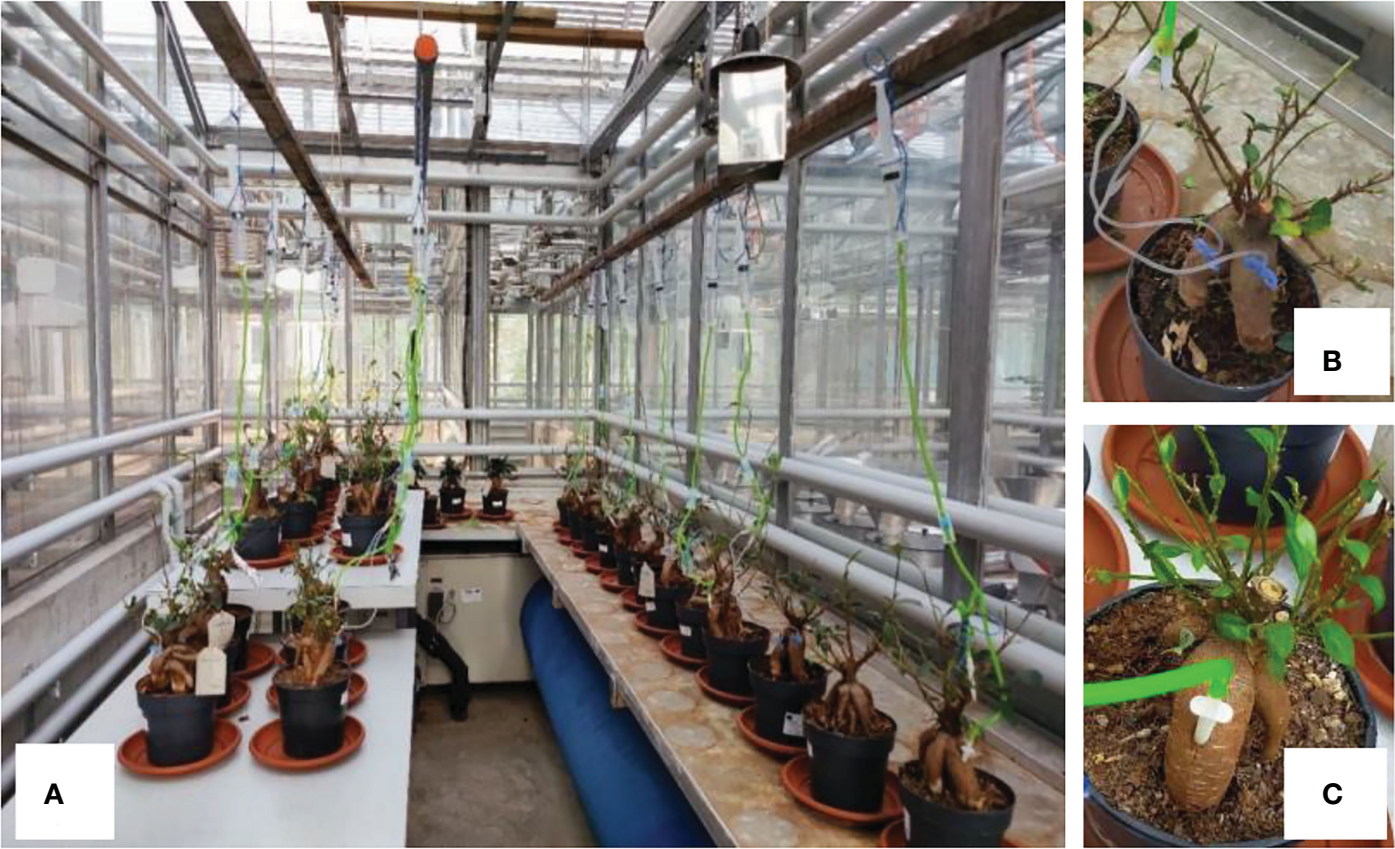
Glucose infusion experiment with *Ficus microcarpa* bonsai plants in WSL greenhouse **(A)**. Each of the three treatments, i.e. phloem infusion **(B)**, xylem infusion **(C)**, and control, had 9 plants. The infusion set consists of a 20 mL-syringe, a 120 cm-long rubber tube with a flow regulator and two thin infusion needles for phloem infusion **(B)**, or one thick plastic needle for xylem infusion **(C)**.

We selected bonsai trees of *Ficus microcarpa* potted in 21 cm (upper diameter) x 18.5 cm (height) x 15 cm (bottom diameter) pots filled with Ficus Mix soil (SYBotanicA, Bemmel, The Netherlands) for a greenhouse experiment at the Swiss Federal Institute for Forest, Snow and Landscape Research WSL, Birmensdorf, Switzerland (**Figure 1A**). Bonsai trees of *F. microcarpa* were selected because they are thick enough with thick xylem and especially thick bark, so that both phloem (**Figure 1B**) and xylem (**Figure 1C**) infusion may be possible. One week prior to sugar infusion treatment, ~85% of the leaves from each of the 27 F*. microcarpa* plants were removed (i.e. only ~15% of leaves in the upper crown were left intact; **Figure 1A**) to limit photosynthesis and thus to create a carbon shortage in plants.

On April 11-12, 2021, the sugar solutions were prepared. We dissolved 10 g ^13^C-labeled D-glucose powder (^13^C_6_, 99 atom % ^13^C, Sigma-Aldrich, Buchs, Switzerland) in 20 mL distilled water as ^13^C-labelled glucose solution (~1:2 for sugar: water). We also dissolved 2 kg non-labelled D-glucose powder (Carl-Roth, Karlsruhe, Germany) in 3 L distilled water as non-labelled glucose solution (~1:1.5 for sugar: water). The δ^13^C value of the non-labelled D-glucose is –26.00‰ ± 0.015‰, and is thus very close to the natural ^13^C abundance (–26.00‰ δ^13^C) in European trees (Taylor et al., 2003; Zeller et al., 2007). This ensures that any ^13^C-labelling effects of the study should thus be solely derived from the ^13^C-labelled glucose. The solution was filtered aseptically in an aseptic platform before infusion.

The infusion set consists of a 20 mL-syringe, a 120 cm-long rubber tube with a flow regulator and two thin infusion needles (size 0.65 x 19 mm; Venofix^®^ Safety G23, Venipuncture set) for phloem infusion (**Figure 1B**), or one thick plastic needle (size 4 x 20 mm; Henan Zhengzhou Green Cube Garden Engineering Co., Ltd., China) for xylem infusion (**Figure 1C**). Before infusion started, we first poured 15 mL non-labeled glucose into each syringe (**Figure 1A**). Nine plants were randomly selected for one of the three treatments, i.e. control, phloem infusion (**Figure 1B**), and xylem infusion (**Figure 1C**) on April 13, 2021. For phloem infusion, the thin, sharp needles were directly inserted into the phloem parallel to the stem surface (**Figure 1B**). For xylem infusion, we first used an electric drill (Bosch Professional GSR 12V-15, Germany) to drill a hole (~10 mm in depth, and 3.5 mm in diameter) in a 45° angle into the stem, and then the thick plastic infusion needle was inserted into that hole (**Figure 1C**), and the whole contact surface was immediately sealed with an adhesive layer (Cementit Universal adhesive, Switzerland). To effectively use the expensive, limited ^13^C-labelled glucose, we used a 1 mL-syringe to inject 1 mL ^13^C-labelled glucose into the lower segment of the rubber tube of each infusion set one day later (April 14, 2021), and the hole on the rubber tube was immediately sealed with an adhesive layer. The initial level of sugar solution in each syringe was marked on the syringe and recorded. The controls were kept intact to measure the natural ^13^C abundance.

**Figure 2:**
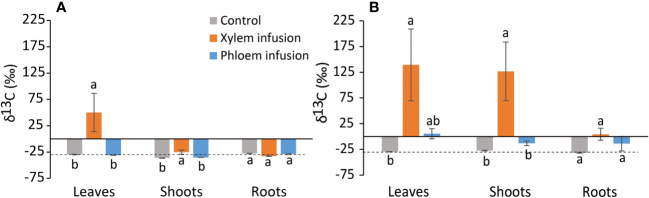
Effects (n = 3 – 5, mean value ± 1 SE) of xylem infusion (orange color) and phloem infusion (blue) with ^13^C-labelled glucose into Ficus microcarpa bonsai trees compared to controls (grey) under greenhouse conditions (see Figure 1). Samples were taken three **(A)** and ten **(B)** weeks after the infusion started. The dashed lines correspond to the average natural ^13^C abundance (~ -26‰ δ^13^C) in European trees (Taylor et al., 2003; Zeller et al., 2007). Different lowercase letters indicate significant differences in δ^13^C values of tissue (i.e., leaves, shoots, or roots) among the treatment xylem infusion, phloem infusion and controls (P < 0.05).

We took leaves, shoots and fine roots (<2 mm in diameter) samples twice during the infusion process: the first sampling date was three weeks later (May 4, 2021) when a visible reduction in the initial level of sugar solution in syringes was observed, and the second sampling date was ten weeks later (June 22, 2021) after infusion started. Leaves, shoots that emerged acropetally above the infusion point and fine roots with white color were collected from each sample plant and immediately transferred and stored at -20°C. We hypothesized that most of the thin white colored roots may be of post-infusion origin, and therefore that they most likely utilized and incorporated the infused exogenous sugars. To collect fine roots, we carefully pulled out the plants from pots, cut the outer white-colored roots, and carefully put the plants back into the pots. Samples were taken only from the bonsai trees, on which the syringe showed a visible lower level of the sugar solution compared to the initial level marked. Only 3 to 5 individuals out of the 9 replicates showed a visible decreased sugar solution level, and the decreasing velocity was normally < 3 mL within the 1^st^ 3 weeks and < 5 mL within the 10 weeks. Thus, the replicates for laboratory and statistical analyses were *n*=3–5 (see **Figure 2**).

### 2.2 Field experiment with adult pine trees

**Figure 3:**
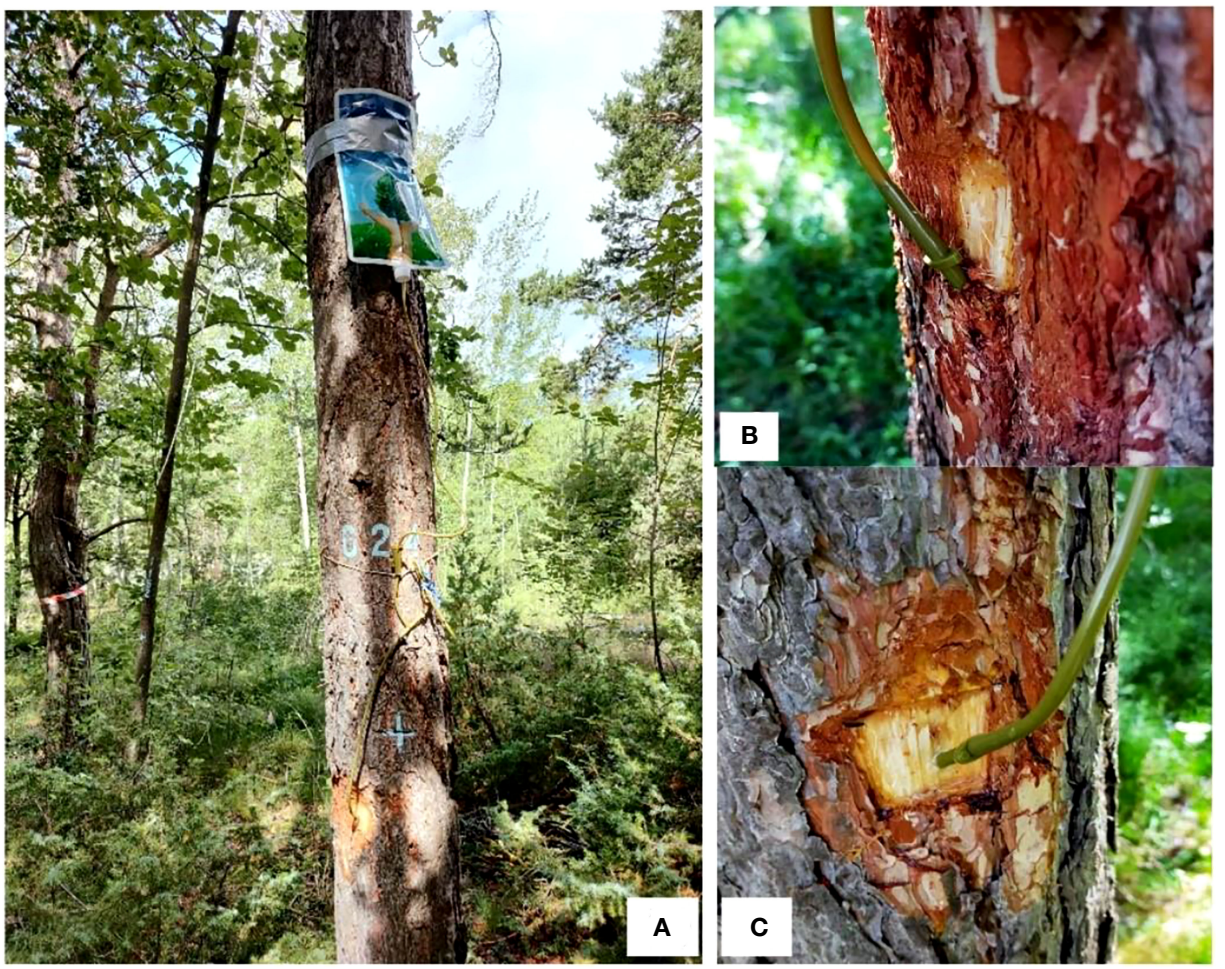
Glucose infusion experiment with adult Scots pine (Pinus sylvestris) trees **(A)**, for phloem infusion **(B)** and xylem infusion **(C)** in Pfynwald, Valais, Switzerland. For phloem infusion **(B)**, a hole (~20 mm in depth, and 3.5 mm in diameter) between the bark and xylem, parallel to the stem surface, was drilled with an electric drill, then the thick plastic infusion needle was inserted into that hole. For xylem infusion **(C)**, we also used an electric drill to drill a hole (~20 mm in depth, and 3.5 mm in diameter) in a 45° angle into the stem, and then the thick plastic infusion needle was inserted into that hole.

The field study was carried out in a mature Scots pine (*Pinus sylvestris* L.) forest located in a dry valley Pfynwald (~600 mm precipitation per year; 46° 18′ N, 7° 36′ E, 615 m above sea level), Valais, Switzerland. Eighteen Scots pine trees (~120 years old, ~11 m in height and ~30 cm in diameter at breast height) grown in similar ambient condition were selected, six out of the 18 trees were randomly assigned for one of the three treatments, i.e. phloem infusion (**Figure 3B**), xylem infusion (**Figure 3C**), and intact controls.

The infusion set consists of a 1.5 L tree infusion bag, a 70 cm-long rubber tube and a thick plastic needle (size 4 x 20 mm; Henan Zhengzhou Green Cube Garden Engineering Co., Ltd., China; **Figure 3**). Both the non-labelled and the ^13^C-labelled glucose solutions used here were prepared as those used in the greenhouse experiment described above. Each infusion bag was filled with 150 mL non-labelled glucose solution. Phloem infusion (**Figure 3B**) and xylem infusion (**Figure 3C**) started on June 28-29, 2021. For phloem infusion, we first used an electric drill (Bosch Professional GSR 12V-15, Germany) to drill a hole (~20 mm in depth, and 3.5 mm in diameter) between the bark and xylem, parallel to the stem surface, then the thick plastic infusion needle was inserted into that hole (**Figure 3B**), and the whole contact surface was immediately sealed with an adhesive layer (Cementit Universal adhesive, Switzerland). For xylem infusion, we also used the electric drill to drill a hole (~20 mm in depth, and 3.5 mm in diameter) in a 45° angle into the stem, then the thick plastic infusion needle was inserted into that hole (**Figure 3C**), and the whole contact surface was immediately sealed with an adhesive layer (Cementit Universal adhesive, Switzerland). The infusion point (position) was at the stem height of 80-100 cm (**Figure 3A**). One week later (on July 7, 2021), we used a 1 mL-syringe to inject 0.8 mL ^13^C-labelled glucose solution into the lower segment of each 70 cm-long rubber tube, and the hole on the tube was immediately sealed with an adhesive layer. Thus, the ^13^C-labelled glucose of 0.8 mL was not diluted in the 150 mL non-labelled glucose solution but concentrated in the lower segment of the rubber tube close to the infusion point. The initial level of sugar solution in each infusion bag was marked on the bag and recorded. The controls were kept intact to measure the natural ^13^C abundance.

**Figure 4:**
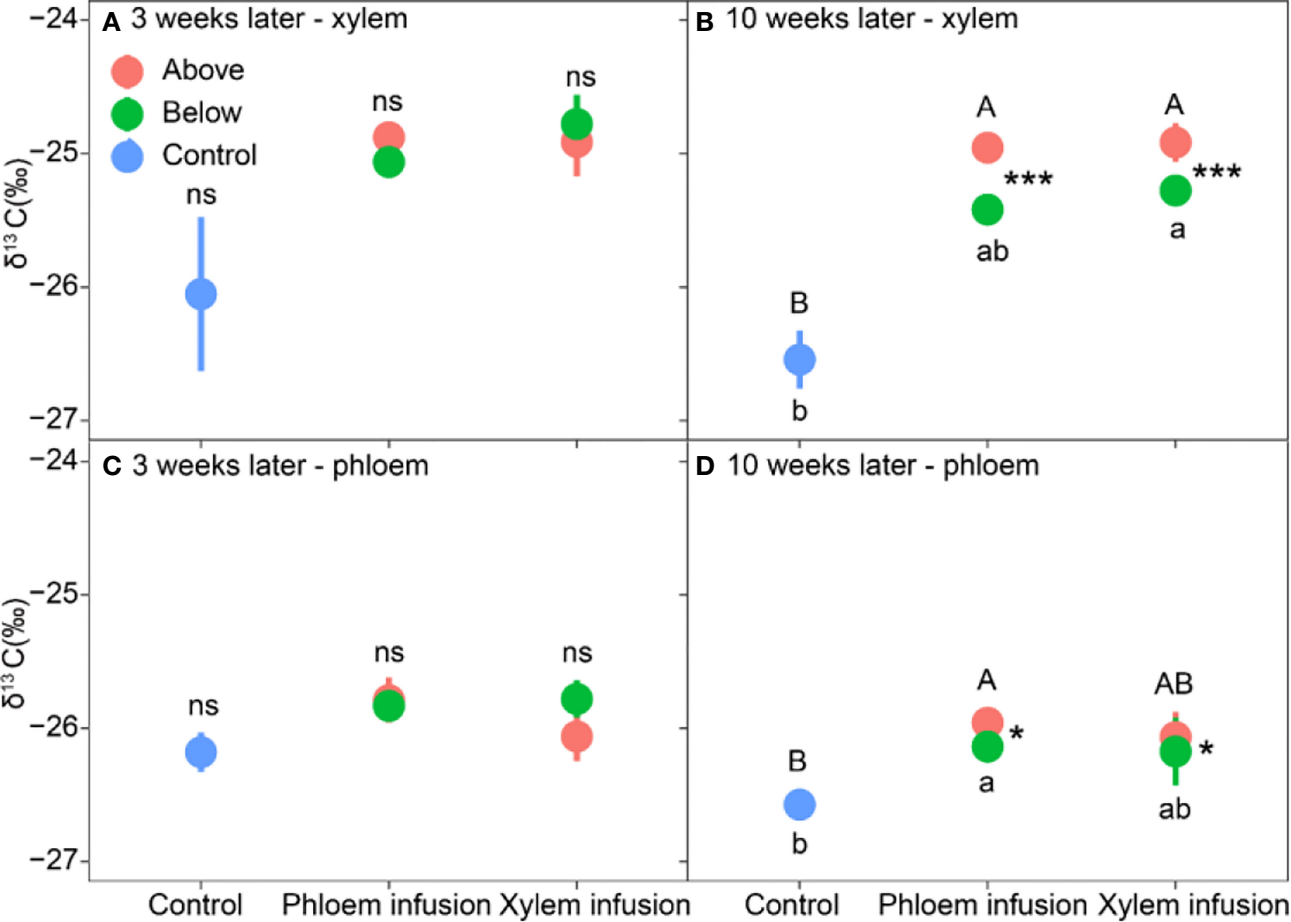
Effects of ^13^C-labelled glucose infusion into phloem and xylem tissues of adult Pinus sylvestris trees compared to controls (n = 3, mean value ± 1 SE). Tissues were collected from positions above (red color) and below (green) the infusion point at the time of three weeks (**A, C**; leaf panel) and ten weeks (**B, D**; right panel) after the infusion started. Note different capital letters indicate significant differences in δ^13^C values among ‘above’-samples and controls, and different lowercase letters indicate significant differences in δ^13^C values among ‘below’-samples and controls. * (P < 0.05) and *** (P < 0.001) denote significant differences in δ^13^C values between ‘above’ and ‘below’-samples within a treatment.

Similar to sampling in the greenhouse experiment described above, we took samples also two times during the infusion process. The 1^st^ sampling date was also three weeks later (July 28, 2021) and the 2^nd^ sampling date was ten weeks later (September 15, 2021) after ^13^C-labelled glucose was added. Samples collected included phloem and the very outer xylem layer, which we assume to be the most active xylem wood. The phloem and xylem samples were taken with treering increment borers (1 cm in diameter, Haglöf Sweden) from the stem height of ~30 cm above (above-sample) and below (below-sample) the infusion point at the 1^st^ sampling date, and of ~50 cm above and below the infusion point at the 2^nd^ sampling date for each infused tree. For the controls, we collected samples at ~1 m (1^st^ sampling) and ~1.3 m (2^nd^ sampling) stem height of each control tree. To obtain materials necessary for ^13^C analysis, 3-5 cores (~1 cm long each) were collected from each stem height level for each sampling date. The bark was removed, and the phloem and xylem were separately collected. Collected samples were kept in polybags in an ice-box carrier, transported to the laboratory and stored at -20°C. Samples were taken only from the treated trees, on which the infusion bag showed a visible lower level of the sugar solution compared to the initial level marked. Only 3 individuals out of the 6 replicates showed a visible decreased sugar solution level (the decreasing velocity could not be estimated due to lack of scale on the bags), and thus, the replicates for laboratory and statistical analyses were *n*=3 (see **Figure 4**).

### 2.3 Respiration measurement experiment with maple saplings

A third experiment with *Acer pseudoplatanus* young trees (170 – 200 cm in height, 1.0 – 1.5 cm in diameter at breast height) infused with ^13^C-labelled glucose was conducted, to test whether plants use the infused exogenous sugars for respiration to provide energy for physiological processes and growth. Using the same infusion set and methods including sugar solutions mentioned above for the field experiment, 3 maple trees were infused with ^13^C-labelled glucose into xylem, and 3 trees were used as controls. Each infused tree was treated with 1 mL ^13^C-labelled glucose plus 30 mL non-labelled glucose, with the same procedure as the field experiment described above. The infusion started on April 14, 2022. Four weeks after infusion started, we tightly wrapped up the upper crown (incl. leaves and small branches) above the infusion point with a black plastic bag (70 L) connected with an input and an output rubber tube. These tubes were then tightly connected to a G2131‐i isotopic‐CO_2_ gas analyzer (Picarro, USA), to measure the dark respiratory ^13^CO_2_ of the upper crown for 20 minutes each (Picarro, 2011).

### 2.4 Laboratory analysis

The bulk ^13^C content of dried and ground samples was analyzed with an elemental analyzer (IsoEarth, Sercon, Crewe, UK) and isotope ratio mass spectrometer (HS2022, Sercon, Crewe, UK) in the WSL stable isotope lab (https://www.wsl.ch/en/about-wsl/instrumented-field-sites-and-laboratories/laboratories/isotope-laboratory.html). The resulting isotope ratios were converted to δ^13^C in per mil by reference to the international standard VPDB with a precision of better than 0.2‰ (Brooks et al., 2022).

### 2.5 Data analysis

**Figure 5:**
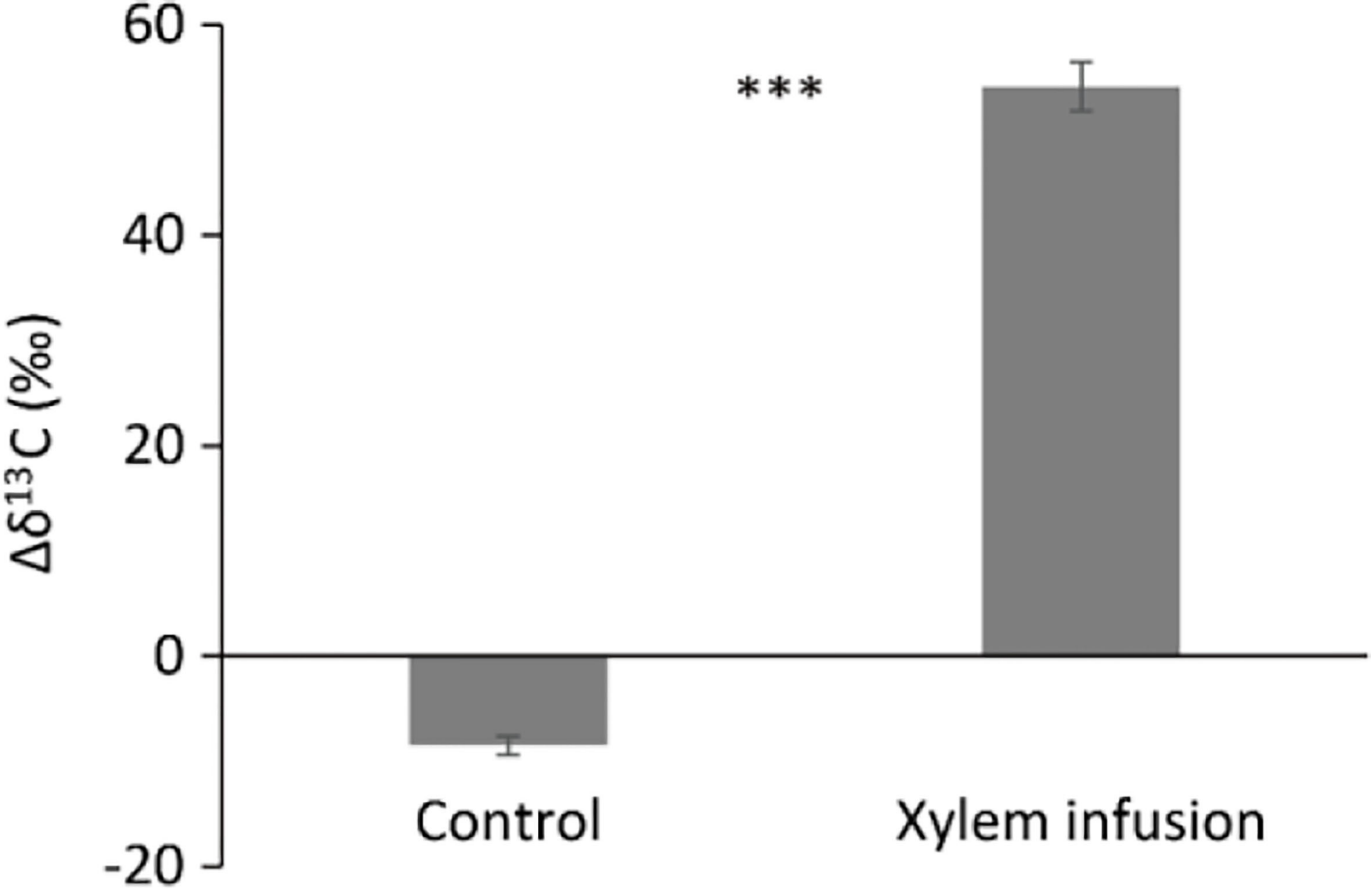
Carbon isotopic composition (δ^13^C) in shoot dark respirated CO_2_ in non-treated *Acer pseudoplatanus* young trees (controls) and trees xylem-infused with ^13^C-labelled glucose (*n* = 3, mean value ± 1 SE). Δδ^13^C (‰) = shoot dark respirated δ^13^C (‰) minus the ambient air δ^13^C (‰). *** (P < 0.001) denote significant differences in Δδ^13^C values between xylem infusion and control.

After the data normality test, we first analyzed the effects of treatment, sampling date (time), and their interaction on tissue δ^13^C levels for the greenhouse experiment with bonsai trees and the field experiment with adult pine trees, respectively. The results showed that the sampling date had significant effects on the tissue δ^13^C levels in 4 out of 7 cases (see **Tables 1**, **2**). We, therefore, tested the treatment effects for each experiment within each sampling date separately. We used one-way ANOVA to analyze the treatment (control, xylem infusion, phloem infusion) effects on δ^13^C values within each tissue type (leaves, shoots, roots) for each sampling date for the greenhouse experiment (**Figure 2**), while we performed two-way ANOVAs to test the effects of treatment (control, xylem infusion, phloem infusion), tissue position (above or below the infusion point), and their interaction on δ^13^C values within each tissue type (xylem, phloem) for each sampling date for the field experiment (**Figures 4**). One-way ANOVA was used to test the difference in the dark respiratory ^13^CO_2_ of the upper crown between the controls and trees infused with ^13^C-labelled glucose (**Figure 5**).

**Table 1:** Effects of treatment (control, xylem infusion, phloem infusion) and sampling date (3 weeks and 10 weeks after the infusion started), and their interaction on δ^13^C values in leaves, shoots and roots of bonsai *Ficus microcarpa* trees, using the linear mixed-effects models.

Sources of variation	df	Leaves	Shoots	Roots
Treatment (T)	2	8.5589 (**0.005**)	7.6523 (**0.007**)	2.1124 (0.164)
Sampling time (S)	1	2.5085 (0.139)	10.1942 (**0.008**)	6.9836 (**0.021**)
T * S	2	0.9462 (0.415)	5.7209 (**0.018**)	3.7049 (*0.056*)

F- and P-values (in brackets) were given, and P < 0.05 is highlighted in bold, and 0.05 < P < 0.10 is shown as marginally significant effect in italics.

**Table 2:** Effects of treatment (control, xylem infusion, phloem infusion) and sampling date (3 weeks and 10 weeks after the infusion started), and their interaction on δ^13^C values in xylem (sampled above and below the infusion point) and phloem (above and below the infusion point) of adult *Pinus sylvestris* trees, using the linear mixed-effects models.

Sources of variation	df	‘Above-’ xylem	‘Below-’ xylem	‘Above-’ phloem	‘Below-’ phloem
Treatment (T)	2	15.9837 (**0.000**)	12.6030 (**0.001**)	5.9558 (**0.016**)	5.4733 (**0.021**)
Sampling time (S)	1	0.6878 (0.423)	4.1222 (*0.065*)	2.4827 (0.141)	10.4388 (**0.007**)
T * S	2	0.4332 (0.658)	0.0439 (0.957)	0.8742 (0.442)	0.0680 (0.935)

F- and P-values (in brackets) were given, and P < 0.05 is highlighted in bold, and 0.05 < P < 0.1 is shown as marginally significant effect in italics.

## 3 Results

### 3.1 Greenhouse experiment

Infusion treatment significantly affected leaf δ^13^C values (P = 0.005) but not root δ^13^C values (P > 0.05), while the infusion treatment interacted with sampling date to significantly affect shoot δ^13^C (P = 0.018; **Table 1**). Across the experiment period, the δ^13^C values in the control plants corresponded to the average level of the natural ^13^C abundance of ~ -26‰ (δ^13^C) in European trees (**Figures 2A, B**). Three and ten weeks after the start of the xylem infusion, δ^13^C values significantly increased in leaves and shoots but not in roots, compared to controls (**Figures 2A, B**). Compared to control plants, phloem infusion did not change the overall δ^13^C level in bonsai trees three weeks after the infusion started (**Figure 2A**), but it tended to increase the tissue δ^13^C level ten weeks after infusion started (**Figure 2B**).

### 3.2 Field experiment

Infusion treatment significantly affected the δ^13^C values in both xylem and phloem collected from both above and below the infusion point (all P < 0.05; **Table 2**). Only “below-” phloem δ^13^C significantly varied with time (P = 0.007; **Table 2**), and ‘below-’ xylem δ^13^C was marginally significantly affected by time (P = 0.065; **Table 2**). Three weeks after the infusion started, both xylem and phloem infusion tended to increase (P > 0.05) the δ^13^C values in xylem and phloem tissue below and above the infusion point compared to controls (**Figures 4A, C**). Ten weeks after the infusion started, the xylem and phloem δ^13^C values did not differ between phloem and xylem infusion (**Figures 4B, D**), but both xylem and phloem infusion increased or tended to increase the δ^13^C values in xylem and phloem tissues taken from the positions both above and below the infusion position, compared to the controls (**Figures 4B, D**). Moreover, ten weeks after the infusion started, the ‘above-’ xylem and ‘above-’ phloem tissues had significantly higher δ^13^C values than the respective ‘below-’ xylem and ‘below-’ phloem tissues for both phloem and xylem infusion (**Figures 4B, D**).

### 3.3 Respiration experiment

Compared to controls, xylem infusion of ^13^C-labelled glucose highly significantly increased the Δδ^13^C values of shoot dark respiration (**Figure 5**), indicating that exogenous sugar was metabolized and used by trees for respiration to produce adenosine triphosphate (ATP) for the physiological processes and tree growth.

## 4 Discussion

### 4.1 Trees take up, translocate, and metabolize infused exogenous sugars

Our results clearly demonstrated that the infused exogenous sugars were taken up and translocated to above- and belowground tissues within a tree (**Figures 2**, **4**), and the respiration experiment confirmed that the exogenous sugars were further metabolized and respired, potentially to provide energy for the physiological processes and tree growth (**Figure 5**).

The present study aimed to develop a method for slowly, longer-lasting addition (i.e. infusion) of exogenous sugars to stressed trees. So far there have been only very few limited (probably 3 only) previous experiments with trees for sugar addition (McLaughlin et al., 1980; Iglesias et al., 2003; Martínez-Trinidad et al., 2009) mentioned above in the section introduction. Those studies, unlike the present study, attempted to fast inject sugars into trees through a hole drilled into a tree trunk or a trough drilled around a tree trunk, and filled with sugar solutions (McLaughlin et al., 1980; Iglesias et al., 2003; Martínez-Trinidad et al., 2009). They all, however, indicated that trees seemed to accept the exogenous sugars. For instance, McLaughlin et al. (1980) introduced ^14^C-labelled sucrose into the stem of a *Quercus alba* tree (~15 cm in diameter at breast height) and subsequently detected and measured the ^14^CO_2_ respiration from soils around that tree, indicating that the ^14^C-labelled sucrose was accepted by the tree and downward allocated to the roots. Iglesias et al. (2003) injected sucrose into stems of *Citrus unshiu* trees and found that sucrose supplementation promoted citrus fruit set by >10%. Martínez-Trinidad et al. (2009) found that trunk sugar injection decreased the stress and improved the growth and vitality of mature (16-20 cm in diameter at breast height) *Quercus virginiana* urban trees under stressful conditions. Sugar injection into crop plants also generally led to increased yield and stress-tolerance (Boyle et al., 1991a; Boyle et al., 1991b; Ma et al., 1994; Tarpley et al., 1994; Hayati et al., 1995; Ma et al., 1995; Zhou and Smith, 1996; Zhou et al., 1997; Abdin et al., 1998; Zhou et al., 2000; Khan et al., 2017). Sorochan (2002) stated that exogenous sugars added can mix with the endogenous carbohydrate pool and be utilized for metabolic processes. Our study suggests that the newly developed methodology will open up new avenues for research on the whole plant C balance and its regulation, and have important implications for practice (e.g. providing additional sugars to historic and landmark trees suffering from severe damage) and future research areas.

It is also worthy to mention that, in the field experiment carried out in the Swiss dry valley Pfynwald (~600 mm precipitation per year), samples taken on September 15 (ten weeks after the infusion started) showed somewhat lower δ^13^C levels than those taken on July 28 (three weeks) (**Figures 4B, D**). For instance, the control trees showed higher δ^13^C levels in tissues sampled on July 28 (-26.1‰ – -26.2‰; **Figures 4A, C**) than those in tissues sampled on September 15 (-26.5‰ – -26.6‰; **Figures 4B, D**). The higher δ^13^C in July (less negative) is a result of summer drought-induced stomatal closure, and the somewhat lower δ^13^C in September is related to decreased temperature and improved soil water conditions, and thus increased openness of stomata (Ehleringer et al., 1992; Eilmann et al., 2010; Timofeeva et al., 2017).

Reduced stomatal conductance or stomatal closure in response to drought stress in July led to reduced CO_2_ concentration in intercellular cavities of leaves (*C*_i_) (Schulze, 1986), relative to atmospheric CO_2_ concentration (*C*_a_), and thus to decreased leaf (*C*_i_/*C*_a_), which have been found to be closely correlated with increases (less negative values) in δ^13^C of newly formed photosynthates (Brugnoli et al., 1988; Farquhar et al., 1989).

### 4.2 Xylem infusion vs. phloem infusion

Xylem infusion but not phloem infusion led to increased tissue δ^13^C levels in the greenhouse experiment with bonsai *Ficus* trees (**Figure 2**), and the respiration experiment with young maple trees showed also highly significant effects of xylem infusion on the Δδ^13^C values of shoot dark respiration (**Figure 5**). These results suggest that xylem infusion is more effective than phloem infusion (see **Figures 4**–**5**), especially for young trees with very thin phloem layer (see **Figures 2**, **5**). This point will be further discussed below in section 4.4. (Advantages and limitations).

Phloem infusion significantly increased δ^13^C values in xylem tissues collected above the infusion point (**Figure 4B**), and it significantly enhanced the δ^13^C values in phloem tissues collected both above and below the infusion point (**Figure 4D**), indicating both upward and downward movement of the infused sugars in phloem. This result is reasonable because phloem consisting of living cells can carry materials both up and down the plant body (Ciffroy and Tanaka, 2018), to allocate photosynthetic carbon products bidirectionally from carbon source (leaves or storage) to sink. In the present study, we might have created a strong C source at the point of C infusion and thus transport in both directions is explainable.

Interestingly, xylem infusion tended to increase the δ^13^C values in roots (**Figure 4B**) and in xylem tissues collected below the infusion point (**Figures 3B, D**), indicating a downward movement of the sugars infused in xylem. Theoretically, this result should not occur since the xylem sap flows unidirectionally upwards (Dixon and Joly, 1894). However, in agreement with our results, bidirectional movement and exchange of many compounds in xylem have also been demonstrated (Gessler et al., 2003; Hijaz et al., 2020). Tattar and Tattar (1999), and Tattar (2009) found both upward and downward movement of xylem-injected mobile dye solution in 8 different tree species with 5 cm to 25 cm in stem diameter at breast height, and they explained those as results caused by the tension break of the water column in the xylem due to the injection wound, which may force the injected solution to move in both upward and downward directions. This explanation may also be valid for our experiment (see **Figure 3**), leading to a ‘dysfunctional’ water movement. Moreover, xylem and phloem streams are neighbored and connected, and thus a diffusive exchange may occur between the two transport systems (Gessler et al., 1998; Gessler et al., 2003; Ciffroy and Tanaka, 2018), which may lead to or contribute to the increased δ^13^C values in roots under xylem infusion (**Figure 2B**) and in xylem below the xylem infusion point (**Figures 4B, D**). On the other hand, the results may also reflect an infusion time-dependent effect. This is when the xylem infusion time is long enough, the δ^13^C signals may move upwards along with the xylem sap flow to leaves firstly, and then downwards along with the phloem sap flow from leaves to roots. It has also been proposed that this two-way movement may be related to the season-dependent leaf transpiration and stress: during the active growing seasons or suitable conditions stem injection may show stronger upward movement, while injections in fall, winter or strong stress (e.g. drought) may result in more downward movement for temperate deciduous trees (Tattar and Tattar, 1999). All these raise the urgent necessity of further studies to explain the theoretical background and the implementation of infusion or injection.

### 4.3 Experiments with young trees vs. adult trees

The labelled δ^13^C values in the young bonsai trees (**Figure 2**, see also **Figure 1**) were much higher (up to +160‰) than those in the adult pine trees (**Figure 4**, see also **Figure 3**), which, certainly, is a result of the dilution effects. We added 1 mL ^13^C-labelled glucose to each infusion set for each small *Ficus* bonsai tree (**Figure 1**), whereas we added only 0.8 mL ^13^C-labelled glucose to each infusion set for each large pine tree (**Figure 3**). The ^13^C concentration is diluted by the large biomass (Li et al., 2001), leading to lower δ^13^C values in tissues of the large pine trees compared to those in the small bonsai *Ficus* trees.

In the greenhouse experiment with small bonsai *Ficus* trees, the leaves had much higher δ^13^C levels than the shoots (**Figure 2B**). This is due to the fact that leaves always have much higher levels of mobile carbohydrates and thus higher δ^13^C levels (Li et al., 2001). Moreover, in a defoliation experiment with *Pinus cembra* trees, Li et al. (2002) found a compensatory mechanism that the defoliated trees prioritize to allocate mobile carbohydrates (thus higher δ^13^C levels) for formation and expansion of new foliage, to compensate for the foliage defoliated and to restore the photosynthesis. In the field experiment with adult pine trees, both phloem infusion and xylem infusion resulted in significantly higher δ^13^C levels in ‘above-’ tissues than ‘below-’ tissues ten weeks after the infusion started (**Figures 4B, D**), indicating a prevailing upward movement of the sugars infused, which has been detailly discussed above in section 4.2 (Xylem infusion vs. phloem infusion).

### 4.4 Advantages and limitations

Here we successfully developed a new technique to infuse exogenous (^13^C-labelled) sugar solution into xylem and phloem tissues of young and mature trees using common infusion equipment for human and animals. However, only 40% (greenhouse experiment) and 50% (field experiment) of the infused trees showed a visible reduction in the initial level of sugar solution after three and ten weeks of infusion, which raises the urgent necessity of further improvement of the infusion method and equipment. Our results indicated that xylem infusion is more feasible and effective than phloem infusion for woody plants (**Figures 4**–**5**). It is very difficult to exactly and successfully insert the very thin infusion needle (see **Figure 1B**) into the very thin phloem layer on the one hand, and on the other hand the pinhole of such very thin infusion needles can very easily be blocked during the infusion process. Compared to the very thin phloem layer, the thick xylem structure allows to be easily operated with infusion on the one side, and on the other side xylem infusion is also more effective than phloem infusion, to infuse a significant amount of sugars into big trees.

The limitations of the present study are that we, using the current equipment, could not control the infusion velocity, and thus do not know the effects of infusion velocity on the infusion effectiveness. Because the price of ^13^C-labelled sucrose is very expensive (> 6200 CHF/g), we did not compare the difference in uptake effectiveness of glucose with sucrose in the present pilot study, although sucrose is the typical form of sugars that is used to translocate carbon and energy in plants. Moreover, the present study did not try to understand the physiological mechanisms for uptake and allocation of the infused sugars and its relations to environmental stress and growth. Our further experiments will consider these limitations for in-depth understanding of the functioning of carbon in stressed trees.

## 5 Prospects and conclusions

The experiments conducted in the present study clearly indicated that trees do take up, translocate and metabolize the exogenous sugars infused, and xylem infusion can be better operated than phloem infusion (especially for young trees), which fully support our three hypotheses (see Introduction). This methodology developed in the present study allows to “feed” trees with sugars, and the tracer allows to study when and where the exogenous carbon is utilized, which can be used to study ecophysiolgoical processes related to carbon starvation or source-sink carbon balances. The method could also be combined with ^18^O, ^15^N, and/or ^2^H-labelling to trace carbon, nutrient, and water movement simultaneously, which will open up new avenues for research on the whole plant resource balance and its regulation in relation to environmental stress, and have great potential for future physiological research areas.

## Data availability statement

The original contributions presented in the study are included in the article/supplementary material. Further inquiries can be directed to the corresponding author.

## Author contributions

M-HL conceived the ideas, designed the methodology, and established the experiments. Y-LZ, YY, and M-HL carried out the experiment. Y-LZ and YY collected and analyzed the data, wrote the manuscript. M-HL led the writing of the manuscript. All authors contributed to the article and approved the submitted version.
